# Innate CD8αα^+^ lymphocytes enhance anti‐CD40 antibody‐mediated colitis in mice

**DOI:** 10.1002/iid3.146

**Published:** 2017-02-20

**Authors:** Aaram A. Kumar, Alberto G. Delgado, M. Blanca Piazuelo, Luc Van Kaer, Danyvid Olivares‐Villagómez

**Affiliations:** ^1^ Department of PathologyMicrobiology and ImmunologyVanderbilt University School of MedicineNashvilleTennesseeUSA; ^2^ Department of MedicineVanderbilt University School of MedicineNashvilleTennesseeUSA

**Keywords:** Granzyme, innate CD8ɑɑ cells, intestinal inflammation, intraepithelial lymphocytes

## Abstract

**Introduction:**

Immune responses in the intestines require tight regulation to avoid uncontrolled inflammation. We previously described an innate lymphocyte population in the intestinal epithelium (referred to as innate CD8αα^+^, or iCD8α cells) that can protect against gastrointestinal infections such as those mediated by *Citrobacter rodentium*.

**Methods:**

Here, we have evaluated the potential contribution of these cells to intestinal inflammation by analyzing inflammation development in mice with decreased numbers of iCD8α cells. We also determined the potential of iCD8α cells to secrete granzymes and their potential role during inflammatory processes.

**Results:**

We found that iCD8α cells play a pro‐inflammatory role in the development of disease in a colitis model induced by anti‐CD40 antibodies. We further found that the effects of iCD8α cells correlated with their capacity to secrete granzymes. We also observed that the pro‐inflammatory properties of iCD8α cells were controlled by interactions of CD8αα homodimers on these cells with the thymus leukemia antigen expressed by intestinal epithelial cells.

**Conclusions:**

Our findings suggest that iCD8α cells modulate inflammatory responses in the intestinal epithelium, and that dysregulation of iCD8α cells effector functions may enhance disease. We propose that one of the mechanism by which iCD8α cells enhance inflammation is by the secretion of granzymes, which may promote recruitment of infiltrating cells into the epithelium.

**Abbreviations**
iCD8α: innate CD8αα^+^
IEL: intraepithelial lymphocyte
TL: thymus leukemia
IEC: intestinal epithelial cell

## Introduction

Intraepithelial lymphocytes (IEL) are a group of immune cells infiltrating the intestinal epithelium that have an intimate relationship with intestinal epithelial cells (IEC). IEL represent a collection of distinct lymphoid cells that can be classified according to their surface marker profile. For example, IEL expressing T cell receptors (TCRαβ^+^ or TCRγδ^+^) can be further subdivided into subsets expressing or lacking expression of the co‐receptors CD4 and CD8αβ 1, 2. A common feature of IEL is the expression of CD8α homodimers. In TCR^+^ cells CD8αα may function as an inhibitor of activation rather than a stimulatory co‐receptor 3. TCR^+^ IEL possess diverse immune functions, encompassing protection from orally transmitted pathogens to maintaining IEC homeostasis 4, 5, 6, 7, 8, 9, 10. Despite the abundant literature concerning TCR^+^ IEL, very little is known about IEL that do not express a TCR. CD3^−^CD7^+^ cells and natural killer cells have been found in the intestinal epithelium 11, 12, and more recently a thorough analysis of innate lymphoid cells (ILC) has indicated the presence of NKp44^+^CD103^+^ and NKp46^+^NK1.1^+^CD160^+^ cells in the IEL compartment of humans and mice, respectively, implicating these cells in inflammatory processes of the intestines 13. Recently, our research group identified a novel population of TCR^−^ IEL characterized by the expression of CD8αα, which we named innate CD8α cells (iCD8α cells) 14. iCD8α cells comprise approximately half of the CD45^+^TCR^−^ IEL population, are only associated with the IEL compartment (i.e. they are not detected in the lamina propria, mesenteric lymph nodes, or Peyer's patches), do not require the transcriptional repressor Id2 for their development (unlike ILC), and their gene expression and cytokine/chemokine profile suggest innate immune functions 14. For example, iCD8α cells are part of the immune response against *Citrobacter rodentium* infection in mice, and a potential role in regulating necrotizing enterocolitis in human neonates has been suggested 14. A recent publication by Ettersperger et al., has confirmed the nature of iCD8α cells 15.

Lymphoid cells of the intestinal epithelium are in a “semi‐activated” state, which allows them to rapidly respond to antigenic and potentially dangerous stimuli derived from the intestinal contents 16. However, because of this feature, the activity of IEL needs to be tightly controlled to prevent unwanted immune responses that may trigger dangerous inflammatory processes. One such regulatory mechanism is mediated by the interaction of CD8α homodimers, which are ubiquitously expressed by a large fraction of IEL, with the thymus leukemia (TL) antigen, a non‐classical MHC molecule expressed by IEC in the colon and small intestine 17, 18. The TL‐CD8αα interaction regulates effector responses of TCR^+^CD8αα^+^ IEL, impacting their proliferation, cytokine secretion, and cytotoxicity 17, 19. Moreover, the TL‐CD8αα interaction influences mucosal immune responses such as protection against *C. rodentium* infection, IL‐17‐mediated immune responses, and exacerbation of colitis in a genetically‐driven mouse model 17, 20.

Considering the importance of the TL‐CD8αα interaction for controlling TCR^+^CD8αα^+^ IEL responses, it is tempting to speculate that this communication also influences iCD8α cells. Indeed, we have shown that TL‐deficient animals have reduced frequencies and numbers of iCD8α cells in the intestinal epithelium, suggesting defective development and/or homeostasis 14. However, the functional status of the remaining iCD8α cells developing in these animals in the absence of TL expression in the intestinal epithelium remains unknown.

In this report, we provide evidence that TL‐deficiency causes increased inflammation in a model of colitis induced with anti‐CD40 antibodies. We found that the observed inflammation was associated with enhanced granzyme secretion by iCD8α cells. Thus, we propose that iCD8α cells promote inflammation via granzyme secretion, which may be regulated by the TL‐CD8αα interaction.

## Results

### TL‐deficient mice exhibit increased susceptibility to anti‐CD40‐induced colitis

**Figure 1:**
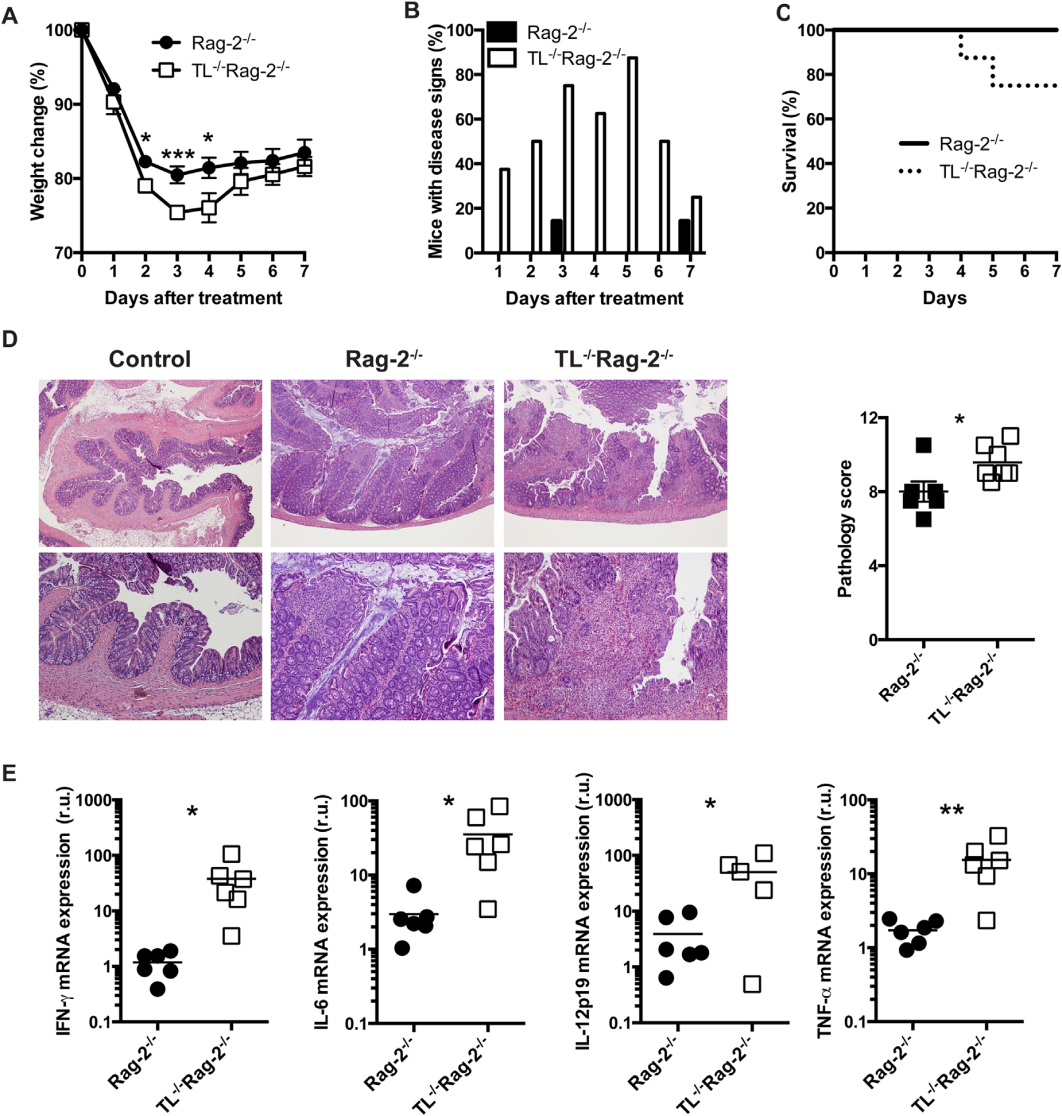
TL‐deficiency renders Rag‐2^−/−^ mice more susceptible to anti‐CD40‐induced colitis. Rag‐2^−/−^ and TL^−/−^Rag‐2^−/−^ mice were treated with 150 μg/mouse of anti‐CD40 antibodies and were monitored daily for (A) weight change, (B) signs of disease such as rectal bleeding, diarrhea and scruffiness, and (C) survival. (D) Colon pathology was determined seven days after treatment as described in the Materials and methods section. Top images, 40× magnification; bottom images, 100× magnification. The graph summarizes the pathology scores. (E) Total colon cytokine mRNA expression was measured by real‐time PCR. Colons from naïve mice were used as reference. The data shown are representative of more than three similar experiments (*n* = 6/experiment). Error bars indicate standard deviation. Student's *t* test was performed. *P < 0.05; **P < 0.01; ***P < 0.005.

To determine the role of iCD8α cells during inflammatory processes we used the anti‐CD40 model of intestinal inflammation. In this model, a single treatment with anti‐CD40 antibodies induces an acute inflammatory process in Rag‐deficient mice 21. Rag‐2^−/−^ mice rapidly lost weight 1 day post‐treatment, whereas mice with reduced numbers of iCD8α cells (TL^−/−^Rag‐2^−/−^ mice) lost considerably more weight especially at days 3 and 4 post‐treatment (Fig. 1A). Weight loss in TL^−/−^Rag‐2^−/−^ mice was accompanied with increased visual signs of intestinal inflammation such as scruffiness, rectal bleeding and diarrhea (Fig. 1B). Also, approximately 20% of TL^−/−^Rag‐2^−/−^mice died around day 4 post‐treatment whereas all Rag‐2^−/−^ mice survived during the course of the experiment (Fig. 1C). Consistent with these results, TL^−/−^Rag‐2^−/−^ mice presented with increased pathology in the colon in comparison to Rag‐2^−/−^ mice (Fig. 1D). To determine the cytokine profile during the early stages of the disease, we analyzed cytokine mRNA expression at 2 days post‐treatment. Both groups of mice produced enhanced levels of pro‐inflammatory cytokine mRNA, especially IL‐12p19, which encodes a subunit of IL‐23, a key cytokine involved in the intestinal inflammation observed in this model 21. However, in accordance with the disease severity observed, TL^−/−^Rag‐2^−/−^ mice presented with a significant increase in the mRNA expression of IFN‐γ, IL‐6, IL‐12p19, and TNF‐α (Fig. 1E).

In summary, our results indicate that TL expression in IEC results in dampened intestinal inflammation in the anti‐CD40 model of colitis.

### iCD8α cells from TL‐deficient mice expand during anti‐CD40‐induced colitis

**Figure 2:**
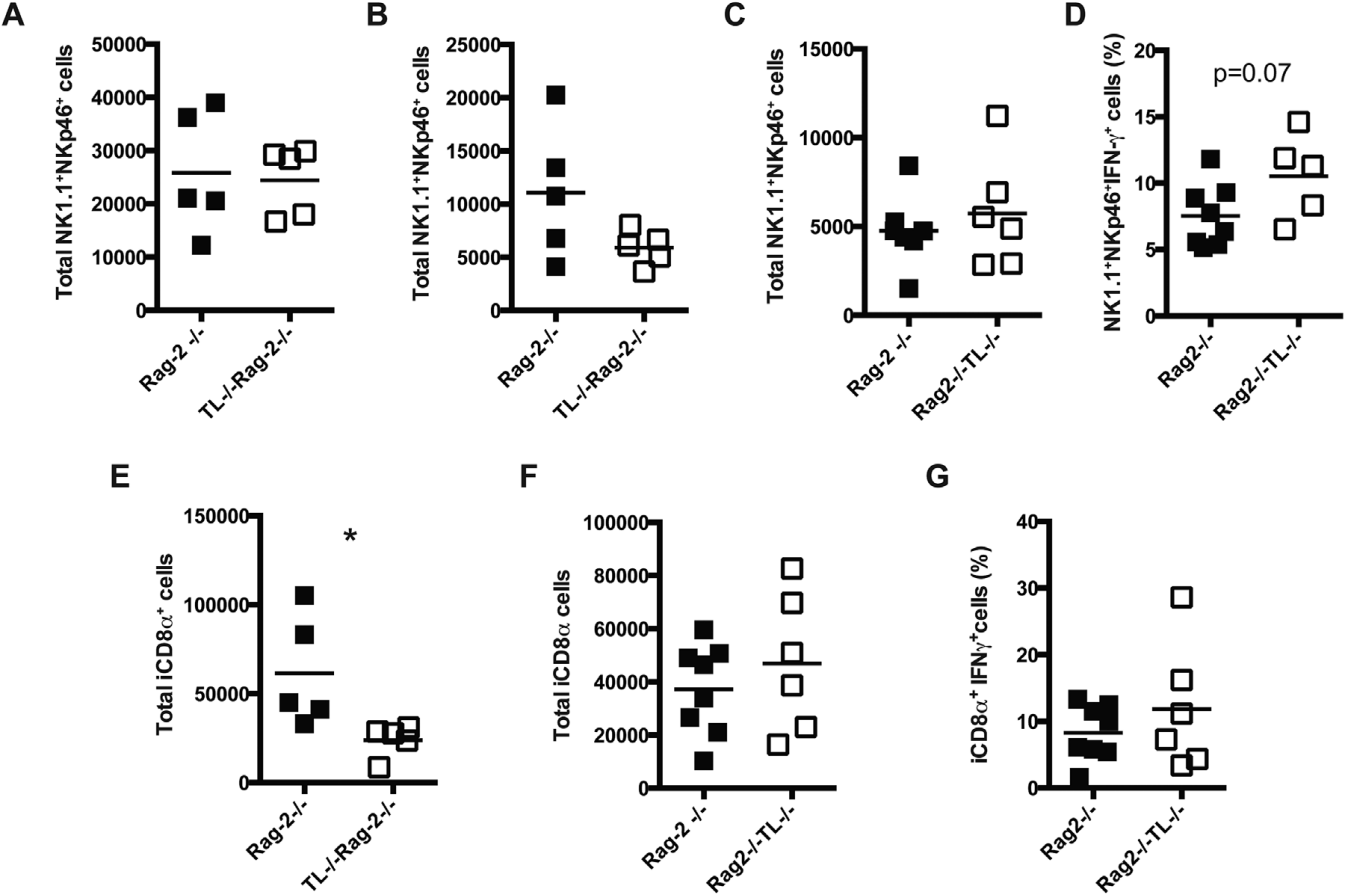
iCD8α cells expand in TL‐deficient mice after anti‐CD40 treatment. Total NK1.1^+^NKp46^+^ IEL numbers in (A) naïve Rag‐2^−/−^ and TL^−/−^Rag‐2^−/−^ mice mice, or animals (B) 1, and (C) 2 days post anti‐CD40 antibodies treatment. (D) Frequencies of NK1.1^+^NKp46^+^ IFN‐γ^+^ IEL from Rag‐2^−/−^ and TL^−/−^Rag‐2^−/−^ mice 2 days post anti‐CD40 treatment. Total iCD8α cell numbers in Rag‐2^−/−^ and TL^−/−^Rag‐2^−/−^ mice after (E) 1 and (F) 2 days treatment with anti‐CD40 antibodies. (G) Frequencies of IFN‐γ^+^ iCD8α cells in Rag‐2^−/−^ and TL^−/−^Rag‐2^−/−^ mice 2 days post‐treatment. The data shown are representative of two independent experiments. Each symbol indicates an individual mouse. Student's *t* test was performed. *P < 0.05.

TL is expressed selectively in IEC and its absence may affect the immune response of CD8αα^+^ IEL, impacting the development of intestinal inflammation. It has been reported that NK1.1^+^NKp46^+^ cells in the IEL compartment promote colon inflammation in the anti‐CD40 model of colitis 13. We therefore, considered the possibility that the difference in colitis between Rag‐2^−/−^ and TL^−/−^Rag‐2^−/−^ mice was due to an increase in the effector functions of NK1.1^+^NKp46^+^ cells in TL^−/−^Rag‐2^−/−^ mice. However, the total cell numbers of NK1.1^+^NKp46^+^ IEL were similar between untreated Rag‐2^−/−^ and TL^−/−^Rag‐2^−/−^mice (Fig. 2A). At day 1 after disease induction there was a reduction in the numbers of NK1.1^+^NKp46^+^ IEL in both groups of mice, probably due to damage to the epithelium; however, cell numbers remained comparable between Rag‐2^−/−^ and TL^−/−^Rag‐2^−/−^ mice (Fig. 2B). Similar results were observed at day two after anti‐CD40 treatment (Fig. 2C). Levels of IFN‐γ production by NK1.1^+^NKp46^+^ IEL were comparable between Rag‐2^−/−^ and TL^−/−^Rag‐2^−/−^ mice (Fig. 2D). These results suggest that, although NK1.1^+^NKp46^+^ IEL are involved in the inflammatory process triggered by anti‐CD40 treatment, these cells likely do not promote increased disease susceptibility in TL^−/−^Rag‐2^−/−^ mice.

In untreated Rag‐2^−/−^ mice the numbers of iCD8α cells range between 100,000–400,000 cells/animal, whereas in naïve TL^−/−^Rag‐2^−/−^ mice iCD8α cell numbers are reduced to a range between 50,000–100,000 cells/animal 14. Analysis of iCD8α cells from Rag‐2^−/−^ and TL^−/−^Rag‐2^−/−^ mice at day 1 after disease induction showed a reduction in the total cell numbers in both groups of mice, but differences in iCD8α cell numbers between Rag‐2^−/−^ and TL^‐/‐^Rag‐2^−/−^ mice remained (Fig. 2E). Interestingly, at day 2 after treatment the numbers of iCD8α cells in TL^−/−^Rag‐2^−/−^ mice increased in comparison to day 1, reaching similar levels to those observed in Rag‐2^−/−^ mice, suggesting that the remaining iCD8α cells in TL^−/−^Rag‐2^−/−^ mice expanded in response to the inflammatory process (Fig 2F). Moreover, we observed similar levels of IFN‐γ production from iCD8α cells derived from TL‐competent or ‐deficient mice (Fig. 2G).

Overall, our results indicate that, during intestinal inflammation caused by anti‐CD40, iCD8α cells but not NK1.1^+^NKp46^+^ IEL expand in TL‐deficient mice.

### iCD8α cells abundantly produce granzymes

**Figure 3:**
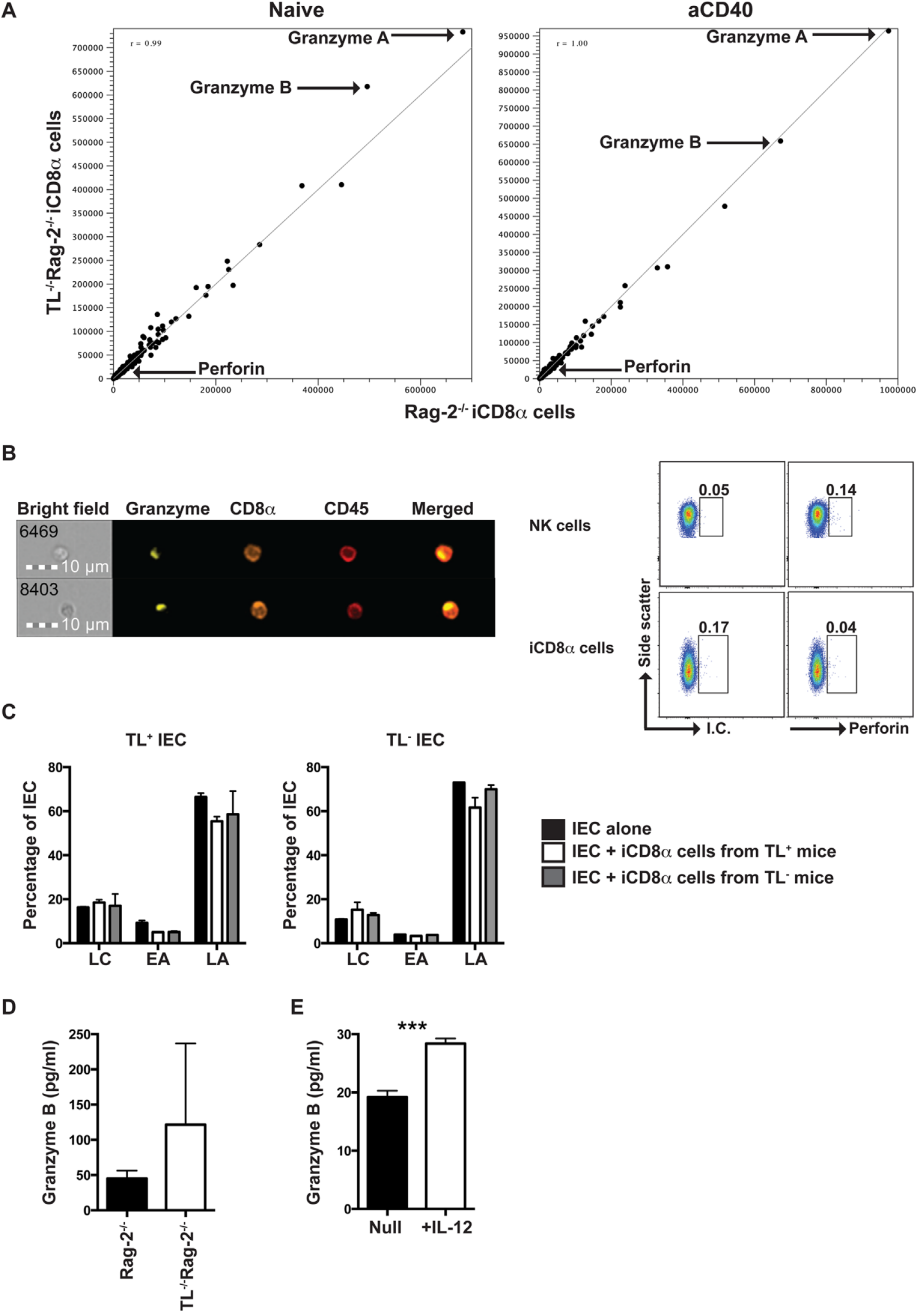
iCD8α cells express large quantities of granzymes. (A) RNA‐seq analysis of iCD8α cells derived from naïve (left) or 1‐day post anti‐CD40 treatment (right) Rag‐2^−/−^ and TL^−/−^Rag‐2^−/−^. Data represent cells derived from at least 10 mice from each group. (B) Granzyme intracellular localization in iCD8α cells derived from naïve Rag‐2^−/−^ mice. The number on the bright field quadrant identifies the acquired cell analyzed. Data show two representative cells of at least 50 cells analyzed with similar results (left image). Spleen NK cells or iCD8α cells were stained for intracellular perforin (right dot plots). (C) IEC derived from TL‐competent or TL‐deficient mice were cultured in the presence or absence of iCD8α cells derived from TL‐competent or TL‐deficient mice for four hours. At the end of the incubation period, IEC were stained for annexin V surface expression. LC, live cells; EA, early apoptosis; LA, late apoptosis. (D) Granzyme secretion by iCD8α cells derived from naïve Rag‐2^−/−^ and TL^−/−^Rag‐2^−/−^ mice. The data shown are representative of two independent experiments. Cells were isolated and pooled from at least 10 mice. (E) Granzyme secretion by IL‐12‐treated or untreated iCD8α cells. The data shown are representative of two independent experiments. Cells were isolated and pooled from at least 10 mice. Error bars indicate standard deviation. Student's *t* test was performed. ***P < 0.005.

Because TL is important for modulating CD8αα^+^ IEL immune responses, and because iCD8α cells from TL‐deficient mice expand during anti‐CD40 treatment, we investigated whether the absence of TL expression in the epithelium promotes a differential gene expression profile in iCD8α cells, imparting these cells with distinct effector functions. However, transcriptome analysis of iCD8α cells isolated from Rag‐2^−/−^ and TL^−/−^Rag‐2^−/−^ mice, either untreated or treated with anti‐CD40 antibodies, failed to reveal differences in gene expression profile (Fig. 3A). However, we observed that the two genes with the highest expression levels were granzyme A and granzyme B, regardless of the TL status (Fig. 3A). Interestingly, in the intestinal epithelium, iCD8α cells are one of the immune cells with greatest granzyme A and B expression, exhibiting higher levels of expression in comparison to CD45^+^CD8α^−^CD11b^+^ myeloid cells and NK1.1^+^NKp46^+^ IEL 14. To determine whether iCD8α cells possess cytotoxic activity, we analyzed the levels of perforin mRNA expression and observed very low levels even after anti‐CD40 treatment (Fig. 3A). Intracellular staining of iCD8α cells showed the presence of granzymes in defined intracellular compartments (Fig. 3B, left images), but perforin was not detected by intracellular staining (Fig. 3B, right plots). Because of the anatomical location of iCD8α cells, it is possible that IEC represent a natural target cell for iCD8α cells. However, when IEC (isolated from TL‐deficient or TL‐competent mice) were cultured in the presence of iCD8α cells (isolated from TL‐deficient or TL‐competent mice), we did not observe an increase in early or late apoptosis that may suggest cytotoxic activity (Fig. 3C). This is in contrast to human NKp44^+^CD103^+^ IEL, the counterpart of mouse NK1.1^+^NKp46^+^ IEL, which degranulate granzymes and perforin in the presence of target cells 13.

Our results show that iCD8α cells are one of the main cells in the intestinal epithelium producing granzymes without detectable cytotoxicity activity.

We reasoned that, if iCD8α cells do not possess cytotoxic activity, they might secrete granzymes in the absence of target cells. To test this hypothesis, we cultured iCD8α cells overnight and then measured granzyme B in the supernatant. iCD8α cells from both Rag‐2^−/−^ and TL^−/−^Rag‐2^−/−^ mice secreted granzyme B at similar levels (Fig. 3D). iCD8α cells constitutively express the IL‐12 receptor and IL‐12 induces production of IFN‐γ in these cells 14. To test whether IL‐12 induces granzyme B secretion, iCD8α cells from naïve Rag‐2^−/−^ mice were cultured in the presence or absence of IL‐12. As shown in Fig. 3E, culture of iCD8α cells in the presence of IL‐12 significantly increased granzyme B production (Fig. 3E).

These results suggest that iCD8α cells are a source of extracellular granzyme B, an effector function that can be induced with IL‐12.

### Intestinal inflammation is associated with granzyme secretion

**Figure 4:**
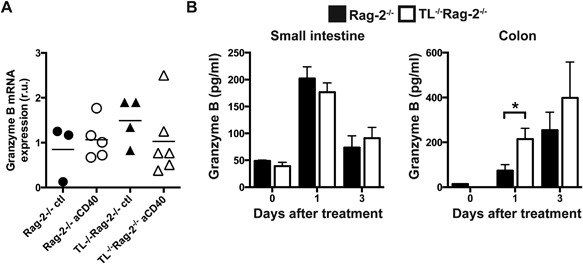
Granzyme levels in the intestines of anti‐CD40‐treated mice. (A) Total granzyme B mRNA expression in the colons of Rag‐2^−/−^ and TL^−/−^Rag‐2^−^ 1 day after treatment with anti‐CD40 antibodies or PBS (ctl). Each symbol represents an individual mouse. (B) Granzyme B levels in the small intestine (left) and colon (right) during the indicated time points after anti‐CD40 treatment. The data shown are representative of 2 independent experiments, (*n* = 5 or 6/experiment). Error bars indicate standard deviation. Student's *t* test was performed. *P < 0.05.

Because of the high levels of granzyme mRNA expression and granzyme secretion by iCD8α cells, we investigated whether these proteases are present during anti‐CD40‐induced intestinal inflammation. For this purpose, we investigated granzyme B mRNA expression in total colon tissue of mice undergoing inflammation in comparison to untreated mice. As shown in Figure 4A, the levels of granzyme B mRNA were comparable between untreated and anti‐CD40‐treated mice, and we detected no difference between the levels of granzyme B mRNA from the colons of Rag‐2^−/−^ and TL^−/−^Rag‐2^−/−^ mice. However, we observed an increase in granzyme B secretion in the small and large intestine at 1 day after anti‐CD40 treatment (Fig. 4B). In the small intestine granzyme levels reached four times what was observed in untreated mice (zero time point). At day three granzyme levels in the small intestine decreased to base line. This pattern was similar between Rag‐2^−/−^ and TL^−/−^Rag‐2^−/−^ mice (Fig. 4B, left). In the colon, granzyme B production 1 day after treatment increased around 100‐fold in Rag‐2^−/−^ mice and almost 200‐fold in TL^−/−^Rag‐2^−/−^ mice over base line. After 3 days, granzyme production in the colons of both groups of mice continued to increase and became indistinguishable between Rag‐2^−/−^ and TL^−/−^Rag‐2^−/−^ mice (Fig. 4B, right).

Overall, our results show that granzyme production correlates with intestinal inflammation. Interestingly, an early boost of granzyme production is observed in the colons of TL‐deficient mice.

### Granzyme release from iCD8α cells correlates with disease outcome

**Figure 5:**
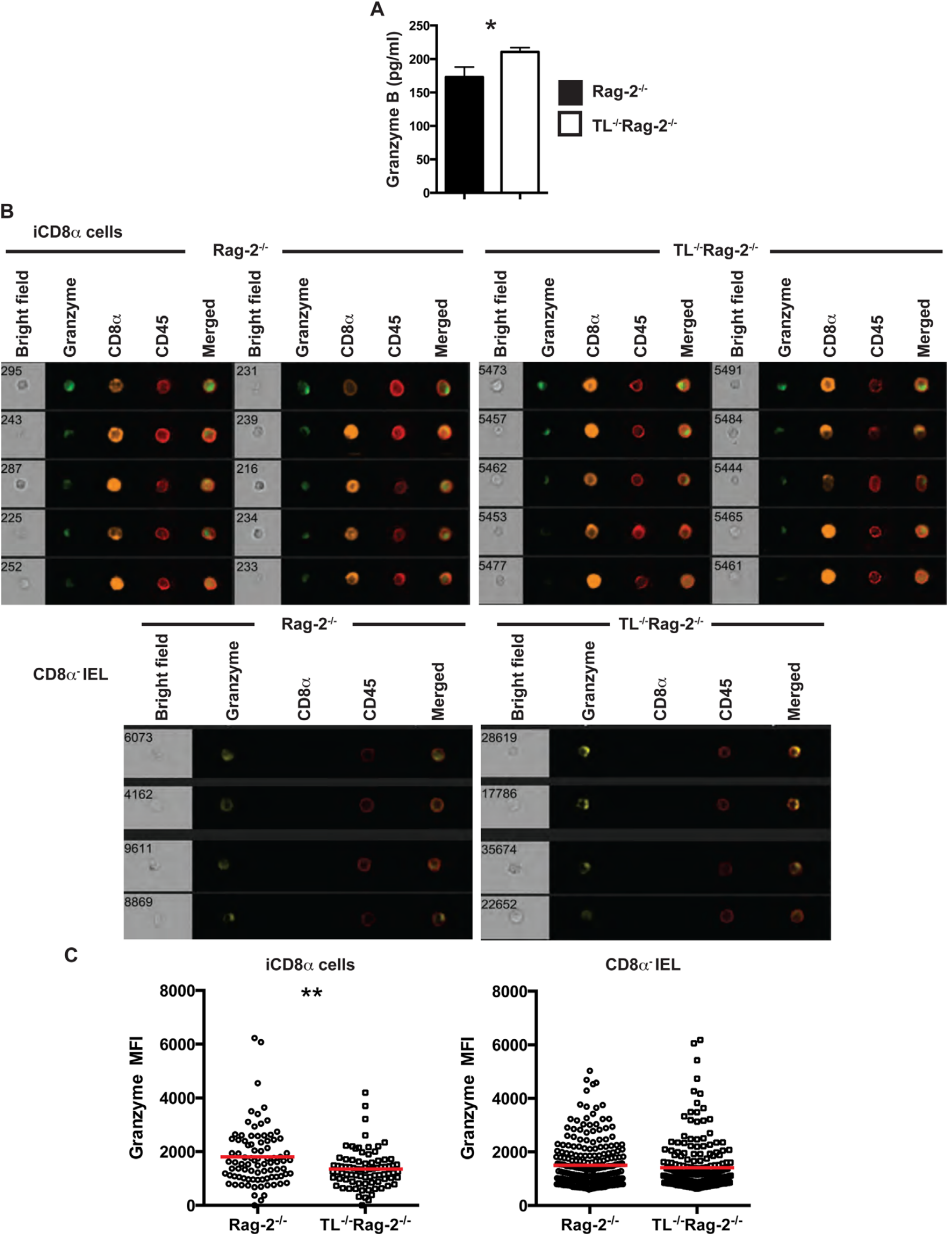
iCD8α cells from TL‐deficient mice secrete increased levels of granzymes. (A) iCD8α cells from anti‐CD40‐treated Rag‐2^−/−^ and TL^−/−^Rag‐2^−/−^ mice (2 days post‐treatment) were incubated overnight and granzyme concentration was determined by ELISA. The data shown are representative of at least two independent experiments, with 5–10 mice per experimental group. Error bars indicate standard deviation. (B) Total IEL from anti‐CD40‐treated Rag‐2^−/−^ and TL^−/−^Rag‐2^−/−^ mice (2 days post‐treatment) were analyzed for intracellular granzyme expression. Top images represent the analysis of iCD8α cells, and the bottom images represent CD45^+^CD8α^−^ IEL. (C) The graphs at the bottom represents a summary of at least 80 iCD8α or CD45^+^CD8α^‐^ IEL cells analyzed for intracellular granzyme intensity from the indicated mice. The data shown are representative of at least three independent experiments and show pooled IEL from at least five mice. Each symbol represents an individual iCD8α cell. Colored bars indicate mean. Student's t test was performed. *P < 0.05; **P < 0.01.

Our results thus far showed that inflammation induced by anti‐CD40 treatment results in granzyme secretion, and that iCD8α cells are a prominent source of secreted granzymes in the intestinal epithelium. To determine whether granzyme B secretion by iCD8α cells correlates with inflammation, we cultured iCD8α cells derived from anti‐CD40‐treated Rag‐2^−/−^ and TL^−/−^ Rag‐2^−/−^ mice and collected the supernatant for analysis of granzyme B levels. We found that iCD8α cells from TL^−/−^Rag‐2^−/−^ mice secreted significantly higher granzyme B levels than cells derived from Rag‐2^−/−^ mice (Fig. 5A). Enhanced secretion of granzymes is expected to be associated with a decrease in intracellular granzyme levels. Indeed, imaging of iCD8α cells derived from mice undergoing inflammation showed a decrease in intracellular granzyme levels in TL^−/−^Rag‐2^−/−^ mice as compared to Rag‐2^−/−^ mice (Fig. 5B and C). In contrast, we observed similar intracellular granzyme levels in the CD8α‐negative IEL fraction from Rag‐2^−/−^ or TL^−/^−Rag‐2^−/−^ mice (Fig. 5B and C). These results suggest that during the first few days of anti‐CD40 stimulation, iCD8α cells are the primary IEL secreting granzymes.

**Figure 6:**
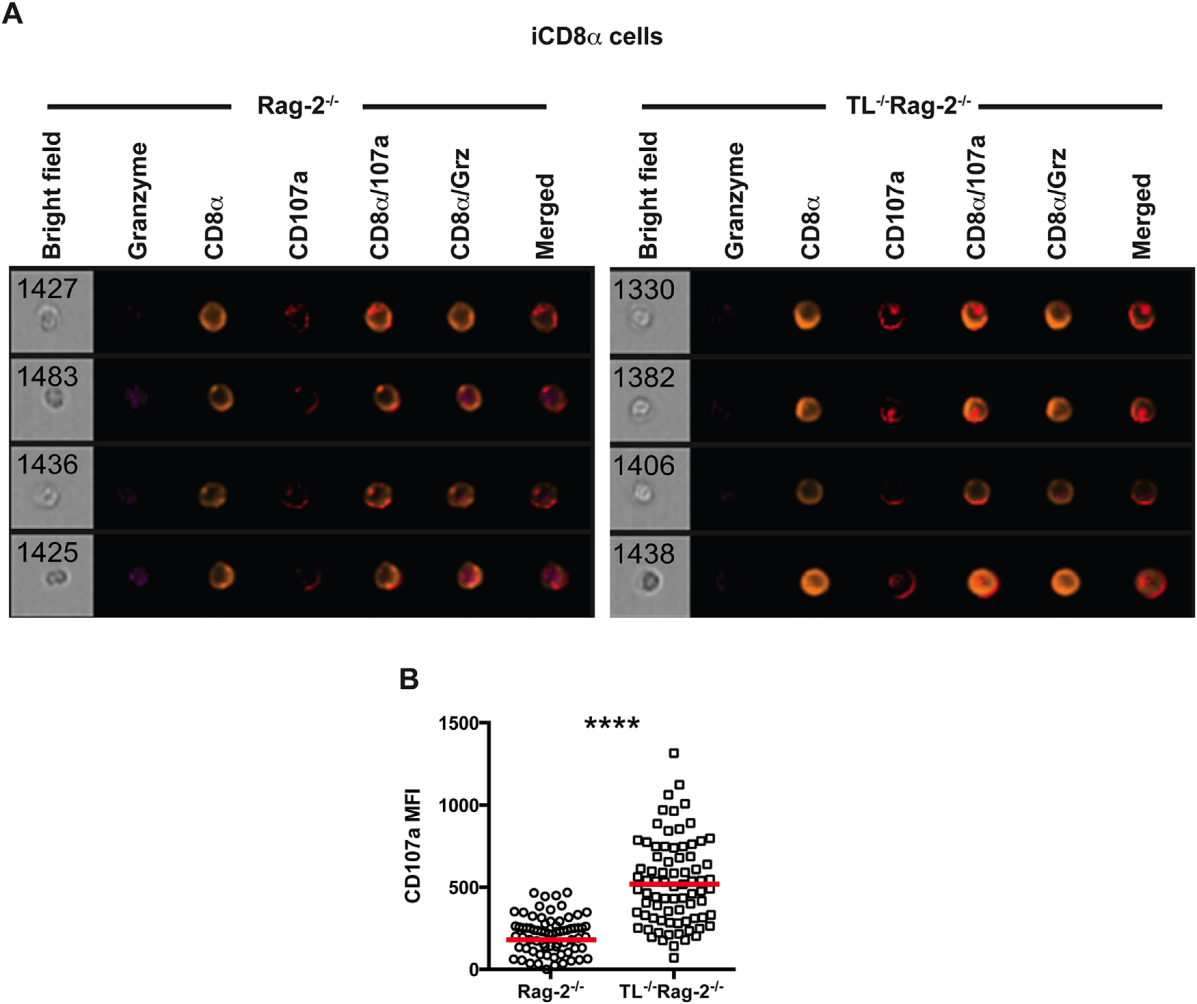
iCD8α cells from TL‐deficient mice express increased surface levels of CD107a. (A) iCD8α cells from anti‐CD40‐treated Rag‐2^−/−^ (left) and TL^−/−^Rag‐2^−/−^ mice (right) were analyzed 2 days post‐treatment for CD107a surface expression. Four representative cells for each group are shown. (B) After acquisition, images were analyzed for CD107a mean fluorescence intensity. The data shown are representative of at least two independent experiments and show pooled IEL from at least three mice. Each symbol represents an individual iCD8α cell. Colored bars indicate mean. Student's *t* test was performed ****P < 0.001.

Reduced intracellular granzyme levels in iCD8α cells from TL^−/−^Rag‐2^−/−^ mice suggest that a higher fraction of these cells have emptied their granzyme‐containing granules. We therefore explored CD107a (Lamp‐1) expression, which is present in the membrane of vesicles directed towards secretion and becomes exposed on the cytoplasmic membrane surface after degranulation 22. Indeed, iCD8α cells from anti‐CD40‐treated mice showed CD107a staining on their surface (Fig. 6A). Concordant with decreased intracellular granzyme staining in iCD8α cells from TL‐deficient mice, we found that these cells expressed significantly higher surface levels of CD107a in comparison to iCD8α cells derived from Rag‐2^−/−^ mice (Fig. 6B).

Our results indicate that, in the absence of TL expression by IEC, iCD8α cells secrete more granzyme B during anti‐CD40‐induced inflammation.

### iCD8α cell‐derived granzyme digest fibronectin

**Figure 7:**
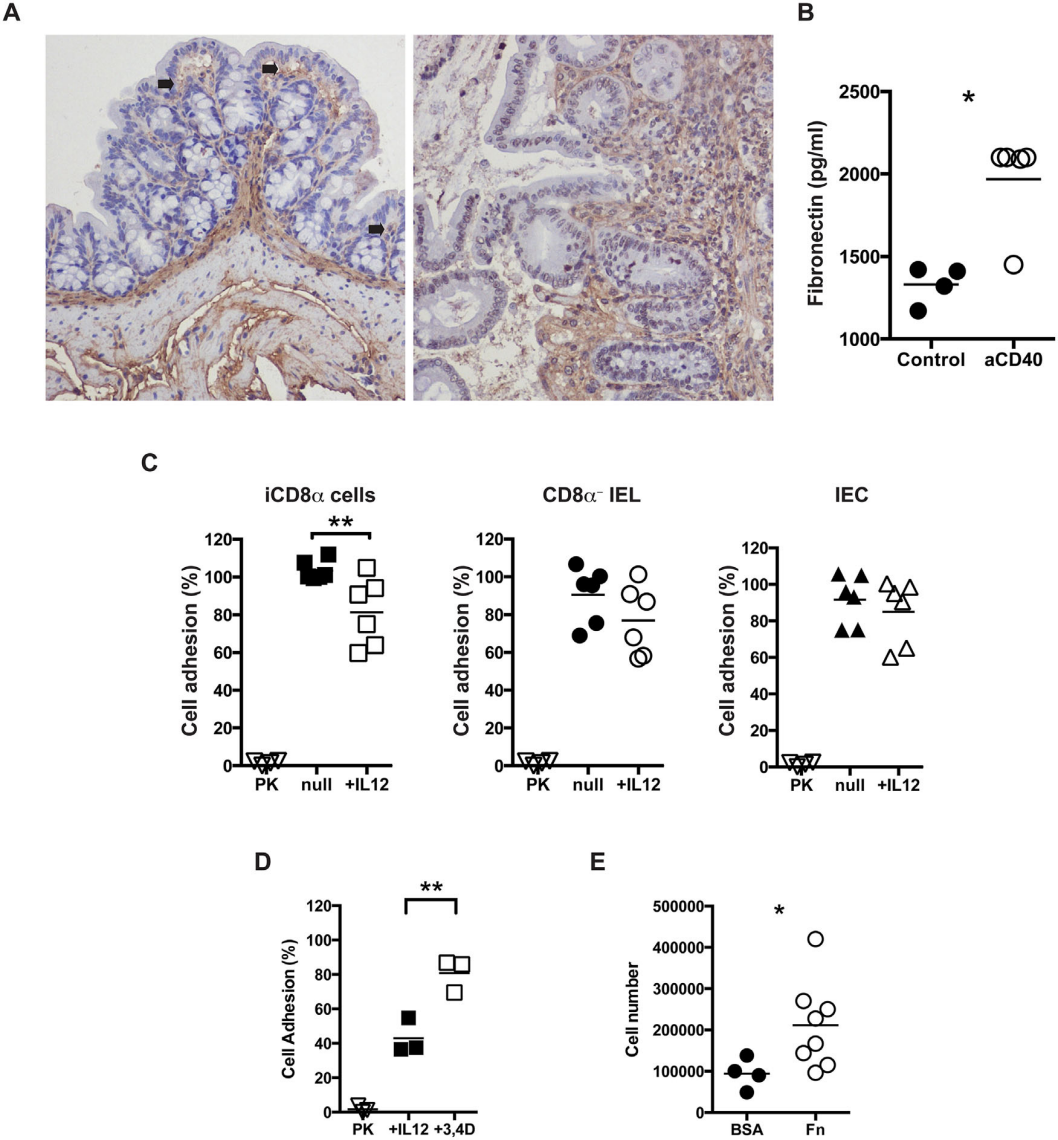
iCD8α cell‐derived granzymes digest fibronectin and promote cell infiltration. (A) Colon fibronectin staining (brown) from naïve (left) or anti‐CD40 treated (right) Rag‐2^−/−^ mice. Arrows indicate areas of close proximity between the epithelium and fibronectin. Magnification, 40×. (B) Soluble fibronectin in total colon cultures of naïve and anti‐CD40‐treated Rag‐2^−/−^ mice (2 days post‐treatment). Each symbol represents an individual mouse. (C) Specific B16 melanoma cell adhesion onto fibronectin‐coated plates treated with supernatants from cultured iCD8α cells (left), CD8α^−^ IEL (middle) and IEC (right) in the presence or absence of IL‐12. PK, proteinase K‐treated wells. (D) 3,4‐dichloroisocoumurin (1 mM) was included in IL‐12‐stimulated iCD8α cell supernatants. (E) Mice were injected i.p. with supernatant of fibronectin or BSA digests. Four hours later, leukocyte recruitment was evaluated by determining total cellularity in the peritoneum cavity. Each symbol represents an individual sample/mouse. Data represents the combination of two independent experiments. Student's *t* test was performed *P < 0.05; **P < 0.01.

Granzymes are known for their role in cell‐mediated cytotoxicity, in which the proteases are delivered into the interior of the target cell where they digest several substrates to initiate cell death. However, as indicated in the previous sections, iCD8α cells may not possess cytotoxic activity. Apart from their role in cytotoxicity, granzymes may have extracellular functions relevant for disease development (referenced in 23). For example, fibronectin present in the extracellular matrix serves as a substrate for granzyme B and is cleaved into several fragments 24, 25. Moreover, it has been proposed that fibronectin fragments may recruit monocytes 26, which may initiate or enhance inflammatory processes. Fibronectin is present in the extracellular matrix of the intestinal submucosa and the lamina propria, reaching the basal side of IEC in naïve mice (Fig. 7A, left). We found that the presence of fibronectin in the intestines increased during anti‐CD40‐induced inflammation (Fig. 7A, left). Soluble fibronectin was also detected in the supernatants of total colon cultures, with increased levels in colons derived from anti‐CD40‐treated mice (Fig. 7B). Considering that iCD8α cells secrete granzyme B during anti‐CD40‐induced inflammation (Fig. 5A), and that fibronectin is present in the intestinal mucosa, we investigated whether iCD8α‐derived granzyme B cleaves fibronectin. Because of difficulties in observing fibronectin digestion in vivo, we adapted an in vitro model system to measure granzyme B digestion of non‐soluble fibronectin 24. In this system, fibronectin‐coated plates were incubated in the presence of supernatants derived from overnight cultures of purified cell populations, followed by measuring B16 melanoma cell fibronectin‐specific cell adhesion. Wells incubated with the potent proteinase K lacked cell adhesion activity, indicating that most of the fibronectin present on the wells was completely digested (Fig. 7C). We then tested whether supernatants from cultures of different IEL populations isolated from Rag‐2^−/−^ mice were capable of digesting fibronectin. The IEL populations we studied were iCD8α cells, CD8α^−^ cells (containing all other IEL including NK1.1^+^NKp46^+^ cells), and IEC. Because IL‐12 induces granzyme release from iCD8α cells, some cell groups were incubated in the presence of IL‐12. Supernatants of iCD8α cells, CD8α^‐^ IEL, and IEC without IL‐12 treatment induced levels of cell adhesion similar to medium alone, representing 100% adhesion (Fig. 7C). However, only supernatants from iCD8α cells incubated in the presence of IL‐12 showed a significant reduction in cell adhesion (Fig. 7C, left). To demonstrate that fibronectin digestion was granzyme B‐dependent, we incubated supernatants from IL‐12‐treated iCD8α cell cultures with 3,4‐dichloroisocoumarin, a granzyme B inhibitor 27, This treatment increased cell adhesion (Fig. 7D), indicating that iCD8α cell‐derived granzyme B is responsible for digesting fibronectin. Interestingly, although non‐stimulated iCD8α cells secrete granzymes, these enzymes do not digest fibronectin (Figure 7C), suggesting that only activated iCD8α cells secrete the active form of these proteases.

To determine whether products derived from the digestion of fibronectin present in the supernatant promote inflammation, we injected supernatants into the peritoneum of Rag‐2^−/−^ mice. As shown in Figure 7E, there was an increase in leukocytes migrating into the peritoneum of mice treated with digested fibronectin in comparison to BSA controls, suggesting that the fibronectin fragments are capable of inducing leukocyte recruitment.

Overall, our results show that iCD8α cell‐derived granzymes are capable of digesting fibronectin, and the digestion products have the potential to recruit leukocytes.

## Discussion

The immune system of the intestinal mucosa constitutes an intricate network of cellular interactions with the ultimate purpose of keeping pathogens at bay, while maintaining overall tissue homeostasis. To avoid excessive and unwanted responses, the mucosal immune system in the gut is tightly regulated. Due to their anatomical location, IEL constitute a critical sentinel barrier to prevent pathogen colonization. During the process of pathogen eradication or other causes of tissue injury, controlled inflammation is important and must be finely tuned to avoid damage to host tissues 28. In this regard, iCD8α cells represent one important cell type of the inflammatory response in the intestines. For example, these cells protect against *C. rodentium* colonization and are believed to be involved in necrotizing enterocolitis 14, Here we report that mice lacking appropriate regulation of CD8αα^+^ IEL via TL 17, 20 exhibit reduced intestinal inflammation induced by anti‐CD40 antibodies than mice where this regulatory mechanism is disrupted. In this experimental system, a single injection of anti‐CD40 induces activation of APC in the absence of T cells (such as in Rag‐deficient mice), which triggers symptoms similar to wasting disease accompanied by intestinal inflammation 21. iCD8α cells are not directly activated by anti‐CD40 treatment because they lack CD40 expression, but are most likely activated by the waves of cytokines triggered following APC activation.

**Figure 8:**
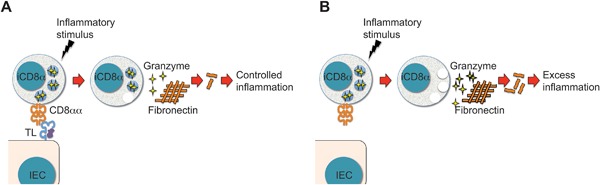
Proposed model for iCD8α cell function in a TL‐competent or −deficient environment. (A) During inflammatory stimuli the TL‐CD8αα interaction regulates the effector function of iCD8α cells. In this scenario, relatively low levels of granzymes are secreted into the epithelium environment, resulting in controlled inflammation. (B) However, when TL is absent, iCD8α cells become dysregulated and inflammatory stimuli may induce these cells to secrete comparatively larger quantities of granzymes, which in turn enhances inflammation.

In the original description of iCD8α cells we reported that activated iCD8α cells secrete cytokines and chemokines characteristic of innate immune responses 14. In this report, we extend this observation to include that iCD8α cells also secrete granzyme B. Granzyme secretion was increased after anti‐CD40 treatment. Interestingly, this process appeared to be perforin‐independent since perforin mRNA levels in unstimulated or activated iCD8α cells were very low and perforin was undetectable by intracellular staining. Moreover, we did not detect cytotoxic activity when iCD8α cells were cultured with target IEC. These observations suggest that iCD8α cell‐derived granzymes possess functions other than lysing target cells. We propose that inflammatory stimuli can activate iCD8α cells, which subsequently secrete granzyme B (and most likely granzyme A as well) into their surroundings, including the extracellular matrix. Secreted granzyme then cleaves proteins such as fibronectin found in the extracellular matrix, and the digestion products may further enhance inflammation (Fig. 8A). In this scenario, iCD8α cell activation would induce a controlled inflammatory process. However, when iCD8α cell activation occurs in the absence of regulatory elements, such as in mice lacking the TL‐CD8αα interaction, there is a burst in secreted granzyme levels during the first 24 hours after disease induction, which may induce an increased inflammatory response (Fig. 8B).

There is increasing evidence indicating that granzymes possess important roles beyond killing target cells in a perforin‐independent manner. For example, cells that are not commonly associated with cytotoxicity properties such as Sertoli cells 29, chondrocytes 30, and plasmacytoid dendritic cells 31 express granzymes. Moreover, granzymes can be found in the plasma of healthy individuals, with increased concentrations in the plasma of individuals undergoing CTL activity, such as patients with EBV or HIV infections 32. Other research groups have reported the presence of granzymes in the synovial fluid of patients with rheumatoid arthritis, in the bronchoalveolar lavage of asthmatic individuals, and in the cerebrospinal fluid 33, 34, 35. Therefore it is conceivable that iCD8α cells represent a source of extracellular granzymes in the intestines.

It has been known for over two decades that a significant proportion of TCR^+^ IEL, including TCRαβ^+^ and TCRγδ^+^ cells, expresses granzymes and perforin, and that under redirected cytotoxicity assays, these cells can lyse target cells 36. Even CD4^+^ IEL that upregulate CD8αα at their surface express granzymes and possess cytotoxic properties 37, 38. Moreover, human NKp44^+^CD103^+^ IEL are also believed to possess granzyme‐ and perforin‐dependent cytotoxic activity 13. In an environment like the intestinal epithelium that is continuously exposed to pathogens and commensal organisms, a full army of granzyme‐competent cells with cytotoxic potential may be beneficial to clear infected cells. However, secretion of granzymes by non‐cytotoxic IEL populations, such as iCD8α cells, may provide a secondary level of host protection. For example, detection of danger signals by iCD8α cells may result in granzyme release into the extracellular matrix to initiate or amplify an inflammatory cascade. Granzyme substrates such as vitronectin, fibrinogen, laminin, and fibronectin are present in the extracellular matrix 24, 25. The relevance of granzyme substrates in the extracellular matrix is accentuated by reports indicating that the digestion products of fibronectin have pro‐inflammatory properties and can recruit peripheral blood monocytes 23, 26, 39. Thus, a potential role for iCD8α cells would be to initiate or enhance inflammation by secreting granzymes, with subsequent digestion of extracellular matrix proteins.

It is important to note that the results provided herein do not establish a direct link between iCD8α cell granzyme secretion and enhancement of intestinal inflammation. Also, our results do not discard the possibility that other IEL or other cell types present in the intestinal epithelium secrete granzymes. However, our results provide a working hypothesis for future research related to iCD8α cell biology and their role in intestinal inflammation.

## Materials and Methods

### Mice

Rag‐2^−/−^ mice on the C57BL/6 background were obtained from the Jackson laboratories and maintained in our colony. TL^−/−^Rag‐2^−/−^ mice on a C57BL/6 background have been previously described 20. All mice were bred and housed under similar conditions. The Institutional Animal Care and Use Committee at Vanderbilt University approved all animal procedures on September 9 2015.

### Anti‐CD40‐mediated colitis induction

Eight‐ to ten‐week‐old mice over 18 g of weight were treated i.p. with 150 μg of anti‐mouse CD40 antibody clone FGK4.5 (Bio × Cell, West Lebanon, NH, USA). Mice were weighed prior to injection and every day after treatment. Mice were monitored daily for signs of disease such as rectal bleeding, diarrhea and scruffiness. At the final time point of the experiment a portion of the proximal colon was used for pathology scoring as previously reported 21.

### Real‐time PCR

A 0.5 cm proximal colon fragment was homogenized using Trizol (Invitrogen, Carlsbad, CA, USA) and the RNA isolated following conventional procedures. RNA was reverse‐transcribed using the High Capacity cDNA Transcription Kit (Applied Biosystems, MD, USA). For real‐time PCR we used the relative gene expression method 40. Actin served as a normalizer. All primers were purchased from QIAGEN (Germantown, MD, USA).

### IEL isolation

IEL were isolated by mechanical disruption as previously reported 17. Briefly, after flushing the intestinal contents with cold HBSS, the intestines were cut longitudinally and excess mucus removed with a pipet tip. Small pieces of the intestine (∼1 cm long) were cut and shaken for 45 min at 37**°**C in HBSS supplemented with 5% FBS. Supernatant was recovered and passed through a gauze column. Cells were recovered and centrifuged in a 40/70% discontinuous Percoll (General Electric, Pittsburgh, PA, USA) gradient. IEL were recovered at the interface.

### Reagents and flow cytometry

Fluorochrome‐coupled anti‐CD8, ‐CD45, ‐CD103, ‐CD107a, ‐granzyme A, ‐IFNγ, ‐NK1.1, ‐NKp46, ‐perforin, and ‐isotype control monoclonal antibodies were purchased from eBiosciences (San Diego, CA, USA) or BD Biosciences (Franklin Lakes, NJ, USA). Uncoupled polyclonal anti‐fibronectin antibodies were purchased from AbCam. For IFNγ intracellular staining cells were stimulated with PMA/Ionomycin for four hours in the presence of GolgiPlug (BD Biosciences). All staining samples were acquired using a FACSCalibur Flow System (BD Biosciences) and data analyzed using FlowJo software (Tree Star, Ashland, OR). For imaging, cells were stained following conventional procedures and resuspended in at least 1 × 10^6^ cells per 30 μL of staining buffer. Samples were acquired using an Amnis FlowSight instrument (Millipore, Billerica, MA, USA), and data analyzed using the IDEAS software (Millipore).

### Cell lysis assay

Purified iCD8α cells from Rag‐2^−/−^ or TL^−/−^Rag‐2^−/−^ mice and IEC (CD45^−^g8.8^+^) also purified from Rag‐2^−/−^ or TL^−/−^Rag‐2^−/−^ mice were co‐cultured for four hours and the annexin V surface pattern was determined by staining following standard procedures.

### Protein detection

A ∼0.5 cm piece of colon or small intestine was cultured in a 24‐well plate in RPMI containing 10% fetal calf serum for 24 hours at 37**°**C, 5% CO_2_, and supernatants were collected. FACS‐enriched iCD8α cells were cultured at 1 × 10^5^ cells/well in a 96‐well plate for 24 hours in the presence or absence of 10–20 ng/mL of recombinant IL‐12 (Peprotech, Rocky Hill, NJ, USA), and supernatants were collected. Granzyme B and fibronectin concentrations were detected using an eBiosciences and AbCam (Cambridge, MA, USA) ELISA kit, respectively.

### RNA‐seq analysis

High quality RNA from FACS‐enriched CD45^+^CD103^+^CD8α^hi^ cells (iCD8α cells) derived from naïve or anti‐CD40 treated mice (1 day post treatment) was sequenced at the Vanderbilt Technologies for Advancement Genomics (VANTAGE, Nashville, TN, USA) core with an Illumina HiSeq 2500. RNA data alignment was performed by Top Hat, followed by gene quantification (FPKM) using Cufflinks. Additional read count was generated with HTSeq. Differential expression analysis was carried out with both FPKM and read count‐based methods. Final analysis was performed with CLC Genomics software.

### Fibronectin cell adhesion assay

FACS‐enriched iCD8α cells (CD45^+^CD103^+^CD8α^hi^), CD8α^‐^ IEL (CD45^+^CD103^+^CD8α^−^), and IEC (CD45^−^) were cultured in the presence or absence of recombinant IL‐12 (10–20 ng/mL). After 24 hours supernatants were recovered and immediately placed in a 96‐well plate coated with fibronectin or BSA (R&D systems, MD, USA) and incubated for four hours at 37**°**C with slight shaking. Some wells were incubated with plain media as control wells. Some wells were incubated with 1 mM of 3,4‐dichloroisocoumurin (Santa Cruz Biotechnology, Santa Cruz, CA). After incubation, supernatants were removed and calcein AM (Tocris Bioscience, Minneapolis, MN, USA)‐labeled B16 melanoma cells were added to the wells (1 × 10^5^/well). After brief centrifugation, cells were incubated for 30 min at 37**°**C. Then, “before wash” fluorescence was measured at excitation and emission wavelengths of 485 and 520 nm, respectively. Non‐adherent cells were washed and “after wash” fluorescence was measured. Specific adherence was calculated as follows: Adhesion = (after wash/before wash) ×100. Specific Adhesion = Fibronectin adhesion—BSA adhesion. For normalization: (Sample SA × control SA)/100.

### Leukocyte recruitment

Supernatants from fibronectin or BSA‐coted plates digested with iCD8α cell‐derived granzymes were injected i.p. into Rag‐2^−/−^ mice. Four hours later the flank of the injection site in the peritoneal cavity was rinsed with 1 mL PBS. Cells were recovered and counted.

### Statistical analysis

Statistical significance between the experimental groups was determined by application of an unpaired two‐tailed Student's *t*‐test or ANOVA. A *P* value < 0.05 was considered significant.

### Human subjects

Human subjects were not used in this study and therefore an ethical approval is not required for this work.

## Conflicts of Interest

None declared.
